# Proteomic Analysis of Pre-Invasive Serous Lesions of the Endometrium and Fallopian Tube Reveals Their Metastatic Potential

**DOI:** 10.3389/fonc.2020.523989

**Published:** 2020-12-15

**Authors:** Mitchell Acland, Georgia Arentz, Max Mussared, Fergus Whitehead, Peter Hoffmann, Manuela Klingler-Hoffmann, Martin K. Oehler

**Affiliations:** ^1^ Adelaide Proteomics Centre, School of Biological Sciences, The University of Adelaide, Adelaide, SA, Australia; ^2^ School of Mathematical Sciences, The University of Adelaide, Adelaide, SA, Australia; ^3^ Cytopathology Department, Clinpath Pathology, Adelaide, SA, Australia; ^4^ Future Industries Institute, Mawson Lakes Campus, University of South Australia, Adelaide, SA, Australia; ^5^ Department of Gynaecological Oncology, Royal Adelaide Hospital, North Terrace, Adelaide, SA, Australia; ^6^ Robinson Research Institute, Discipline of Obstetrics and Gynaecology, Adelaide Medical School, The University of Adelaide, SA, Australia

**Keywords:** proteomics, serous tubal intraepithelial carcinoma, endometrial intraepithelial carcinoma, serous endometrial carcinoma, high grade serous ovarian carcinoma

## Abstract

Serous endometrial cancer (SEC) and high grade serous ovarian cancer (HGSOC) are aggressive gynecological malignancies with high rates of metastasis and poor prognosis. Endometrial intraepithelial carcinoma (EIC), the precursor for SEC, and serous tubal intraepithelial carcinoma (STIC), believed to be the precursor lesion for HGSOC, can also be associated with intraabdominal spread. To provide insight into the etiology of these precancerous lesions and to explore the potential molecular mechanisms underlying their metastatic behavior, we performed a proteomic mass spectrometry analysis in a patient with synchronous EIC and STIC. Through histological and molecular identification of precancerous lesions followed by laser capture microdissection, we were able to identify over 450 proteins within the precancerous lesions and adjacent healthy tissue. The proteomic analysis of STIC and EIC showed remarkable overlap in the proteomic patterns, reflecting early neoplastic changes in proliferation, loss of polarity and attachment. Our proteomic analysis showed that both EIC and STIC, despite being regarded as premalignant lesions, have metastatic potential, which correlates with the common presentation of invasive serous gynecological malignancies at advanced stage.

## Introduction

Endometrial cancer (EC) is the 6^th^ most common cancer in women worldwide and is the most common gynecological malignancy (1). Despite significant advances in early detection (2), molecular subtyping (3, 4), and new or improved treatment regimens (5), the relative survival of patients with EC has declined in recent times (6, 7).

Serous endometrial carcinoma (SEC) is a highly aggressive malignancy (8). It represents only 10% of EC cases (9, 10) but is responsible for 39% of EC related deaths (11) and is frequently diagnosed at late stage when prognosis is poor (9, 10). The current model of SEC development suggests that it evolves from a pre-neoplastic lesion in atrophic endometrium called endometrial glandular dysplasia (EmGD) (12, 13). This lesion then progresses further into endometrial intraepithelial carcinoma (EIC) and, finally, into SEC (14).

EmGD is characterized by loss of cell polarity, nuclear atypia, and nuclear hyperchromasia (12). It has a marked loss of heterozygosity in TP53 and chromosome p1; however, this is to a lesser extent than that seen in EIC and SEC (12). Although quite difficult to identify, nucleomegaly and staining for p53, MIB-1 as well as IMP3 are characteristics of EmGD (14). The connection between EmGD and SEC has been confirmed through identical mutations observed in EmGD and subsequent SEC (15).

EIC was first described in the 1990s (16) and seen to arise almost exclusively in atrophic endometrium and in the context of SEC in a majority of cases (17). EIC exhibit similar features as EmGD but with further nucleomegaly, nuclear irregularity and hyperchromasia (14). However, cases of EIC associated with extrauterine metastasis suggest that EIC may more closely resemble SEC (18, 19). This early peritoneal spread is in stark contrast to non-serous endometrial cancers which usually do not show early peritoneal spread but preferentially invade the myometrium and spread to the lymph nodes (18, 19).

SEC closely resembles other serous cancers of the female genital tract, such as high grade serous ovarian cancer (HGSOC) (4). Both diseases share similar molecular features and clinical properties (20) and, consequently, are treated in a similar way (21, 22).

In recent years the fallopian tube has been identified as the precursor site of HGSOC, specifically serous tubal intraepithelial carcinoma (STIC) (23–27). These lesions are identified histopathologically by a “p53 signature” comprised of strong p53 staining (28), p53 mutations (29), positive γ-H2AX staining (indicating DNA damage) and lack of Ki-67 staining (indicating low proliferation) (30) (**Table 1**). STICs share many genomic features with HGSOC, such as genomic instability (31, 32), and HGSOC has a gene expression profile more similar to the fallopian epithelium than the ovarian surface epithelium (33).

**Table 1:** Morphological and molecular features of precancerous lesions of the gynecological tract.

Location	Precancerous lesion	Morphological features	Molecular features
Endometrium	EmGD	Some nuclear atypia, some nucleomegaly.	Low rates of loss of heterozygosity in TP53 and chromosome P1. p53 mutations in 50% of cells. Staining for P53, MIB1, and IMP3.
	EIC	Extensive nuclear atypia, extensive nucleomegaly, hyperchromasia.	High rates of loss of heterozygosity in TP53 and chromosome P1. p53 mutations in 75% of cells. Strong staining for P53, MIB1, and IMP3.
Fallopian tube	ESTP	No visible morphological features.	TP53 mutations, p53 staining, DNA damage.
	STIC	Hyperchromasia, nucleomegaly.	TP53 mutations, p53 and MIB1 staining, DNA damage, Chromosomal instability.

Results from studies using mouse models have established a connection between STIC and subsequent HGSOC (34, 35). Inactivation of PTEN, p53 and BRCA1/2 in the fallopian tubes of mice resulted in STIC and concurrent HGSOC with ovarian and peritoneal spread (34, 35). However, in the absence of BRCA1/2 inactivation, STIC developed but did not progress to metastatic HGSOC in this mouse model (34).

Cases of HGSOC arising in the absence of STIC (35–37) have also been reported suggesting that other, as yet unidentified, precursor lesions might exist (38). “Early Serous Tubal Proliferations” (ESTP) have been identified as a potential HGSOC precursor. They are found in the fimbria (30), demonstrate DNA damage (30), and are found in non-ciliated cells, which also give rise to STIC (39). A physical and lineage continuity has been demonstrated between ESTP and STIC suggesting that some ESTP give rise to STIC and subsequently to HGSOC (30, 32).

The understanding that HGSOC arises from the fallopian tube in many cases has changed the understanding of serous ovarian cancer. Now serous cancers of the fallopian tube, peritoneum and ovary are thought to share a common origin in the fallopian tube (20). While it is well established that many HGSOC arise from the fallopian tube, it has not been excluded that some serous cancers of the endometrium and ovary may share common origins. For example, Roelofsen et al. (40) suggested that some serous ovarian cancers (SOC) may arise from EIC by showing that they shared TP53 mutations, similar expression of p53, Ki67, estrogen, and progesterone receptors (40). Additionally, Tolcher et al. (20) analyzed 38 patients with SEC and investigated their fallopian tubes. They found STIC, without evidence of tubal metastasis, in 2 of these cases (20).

To better understand the potential link between serous preinvasive lesions of the female genital tract and serous gynaecological cancer, molecular investigations of the precursor lesions of the endometrium and fallopian tube are required. Here, we present the first proteomic analysis of synchronous precancerous lesions of the endometrium and fallopian tubes in a patient without invasive malignancy, by means of mass spectrometry. This precludes the possibility of these premalignant lesions representing metastases from established primary tumors. The analysis of EIC and STIC in this context provides insight into the temporal and mechanistic features of their development and dissemination.

## Materials and Methods

### Sample

Archived formalin-fixed paraffin-embedded (FFPE) fallopian tube and endometrial tissues from a 67-year-old female who had undergone a total abdominal hysterectomy and bilateral salpingo-oophorectomy for endometrial hyperplasia was retrieved for analysis with approval of the Research Ethics Committee of the Royal Adelaide Hospital. The fallopian tube and endometrial tissues were processed using standard procedures, stained with hematoxylin and eosin, and annotated by a pathologist. P53 and MIB1 immunostaining was also performed to confirm the location of precancerous lesions.

### Laser Microdissection and Sample Preparation

FFPE tissues were sectioned at 4-µm thickness, water bath mounted onto PEN membrane slides (Micro-Dissect, Herborn, Germany), and deparaffinized by submersion in xylene for 5 min, following by two 2-min incubations in 100% ethanol, and two 5-min incubations in water. Areas of STIC, EIC and adjacent healthy epithelium were dissected using a Leica AS LCM microscope (Leica Microsystems, Wetzlar, Germany) into 20 μl of 10 mM citric acid buffer (pH = 6) and subjected to heat induced antigen retrieval by incubation at 100°C for 90 min. The solution containing the protein extracts were digested with trypsin gold (Promega, Madison, WI, USA) as described in Mittal et al. (41) using a modified FASP method (42). In brief, protein extracts were mixed with 0.2 ml of 8M urea in 0.1M Tris/HCl, pH 8.5 before being loaded into a 30k Microcon filtration device (Millipore) and centrifuged at 14,000g for 15 min. This step was repeated to ensure the removal of any residual contaminants. Samples were reduced with 5 mM DTT (Roche) for 45 min at room temperature and alkylated with 10mM iodoacetamide (IAA) (GE Healthcare, Little Chalfont, UK) for 30 min at room temperature in the dark followed by centrifugation at 14,000g for 15 min. The protein concentrate was diluted with 0.2mL of 8M urea in 0.1M Tris/HCl, pH 8.5, and spun again at 14,000g for 15 min. This step was repeated twice. Samples were buffered with 10mM NH_4_HCO_3_ and digested with 100ng trypsin gold overnight at 37°C. Peptides were collected by centrifugation of the filter unit at 14,000g for 20 min.

### Nanoflow Liquid Chromatography Tandem Mass Spectrometry

Nanoflow liquid chromatography tandem mass spectrometry (Nano-LC-MS/MS) was performed on each sample in duplicate using an Ultimate 3000 RSLC system (Thermo-Fisher Scientific, Waltham, USA) coupled to an Impact HD™ QTOF mass spectrometer (Bruker Daltonics, Bremen, Germany) *via* an Advance CaptiveSpray source (Bruker Daltonics). Peptide samples were pre-concentrated onto a C18 trapping column (Acclaim PepMap100 C18 75 μm × 20 mm, Thermo-Fisher Scientific) at a flow rate of 5 μl/min in 2% (v/v) ACN 0.1% (v/v) TFA for 10 min. Peptide separation was performed using a 75 μm ID C18 column (Acclaim PepMap100 C18 75 μm × 50 cm, Thermo-Fisher Scientific) at a flow rate of 0.2 μl/min using a linear gradient from 5 to 45% B (A: 5% (v/v) ACN 0.1% (v/v) FA, B: 80% (v/v) ACN 0.1% (v/v) FA) over 130 min, followed by a 20-min wash with 90% B, and a 20-min equilibration with 5% A. MS scans were acquired in the mass range of 300 to 2,200 m/z in a data-dependent fashion using Bruker’s Shotgun Instant Expertise™ method. This method uses IDAS (intensity dependent acquisition speed) to adapt the speed of acquisition depending on the intensity of precursor ions (fixed cycle time), and RT^2^ (RealTime Re-Think) to exclude previously selected precursor ions from undergoing re-fragmentation unless the chromatographic peak intensity of the ion has increased by a factor of 5. Singly charged precursor ions were excluded from acquisition. Collision energy ranged from 23% to 65% as determined by the m/z of the precursor ion.

### Data Analysis

Spectra were analyzed using the MaxQuant software (version 1.5.2.8) with the Andromeda search engine (43) against the UniProt non-redundant human database. The standard Bruker QTOF settings in MaxQuant were used with a mass error tolerance of 40 ppm. The variable modifications of oxidation of methionine and the fixed modification of carbamidomethyl of cysteines were specified, with the digestion enzyme specified as trypsin. The protein false discovery rate (FDR) and peptide spectrum match FDRs were both set to 1% using a target decoy approach, with a minimum peptide length of 7 amino acids (43). Only unique and razor peptides were used when reporting protein identifications.

### Gene Expression Analysis

In order to assess the gene expression levels of the corresponding proteins of interest in early stage I ovarian carcinoma tissues, the dataset of Yoshihara et al. (44) [Gene Expression Omnibus (GEO) Accession GSE12470, http://www.ncbi.nlm.nih.gov/geo/] was considered. From this dataset the expression of EPCAM and CAPS was considered in 8 in early stage I patients compared to 10 healthy peritoneum control tissues. The results were natural log transformed and compared using paired T-tests and p-values < 0.05 were considered significant. Full patient details are available in the Yoshihara et al. (44) manuscript.

To assess the gene expression levels of the corresponding proteins of interest in differing subtypes of EOC, the dataset of Hendrix et al. (45) (GEO Accession GSE6008, http://www.ncbi.nlm.nih.gov/geo/) was considered. From this dataset the expression of TPPP3, SORD and VCAN was investigated in 37 endometrioid, 41 serous, 13 mucinous, and 8 clear cell ovarian carcinoma tissues, and 4 normal control tissues. Transformation of the data and full patient details are available from the Hendrix et al. (45) manuscript. Groups were compared using paired T-tests and p-values < 0.05 were considered significant.

With the aim to assess the gene expression levels of corresponding proteins in early stage 1 endometrial carcinoma tissue, R was used to investigate the data set of Days et al. (46) (GEO Accession GDS4589, http://www.ncbi.nlm.nih.gov/geo/). This this dataset the expression of EPCAM and CAPS were investigated in 79 endometrioid and 12 serous papillary endometrial carcinoma tissues, and in 12 normal control tissues. The results were natural log transformed and compared using paired T-tests. P-values < 0.05 were considered significant. Full patient details are available through the Day’s et al. (46) manuscript.

To evaluate the gene expression levels of corresponding proteins in different subtypes of endometrial carcinoma tissue, R was used to investigate the data set of Kandolth et al. (4) (https://gdc.cancer.gov/node/875). From this data set the expression of TPPP3, SPATA18, ERO1A, SORD and VCAN were investigated in 13 CN high, 15 CN low, 16 MSI hypermutated, and 4 POLE ultra-mutated carcinoma tissues. Transformation of the data set is detailed in Kandolth et al. (4). Groups were compared using paired T-tests and p-values < 0.05 were considered significant. Full patient details are available in the Kandolth et al. (4) manuscript.

## Results

### Histological Analysis Identifying STIC and EIC

**Figure 1:**
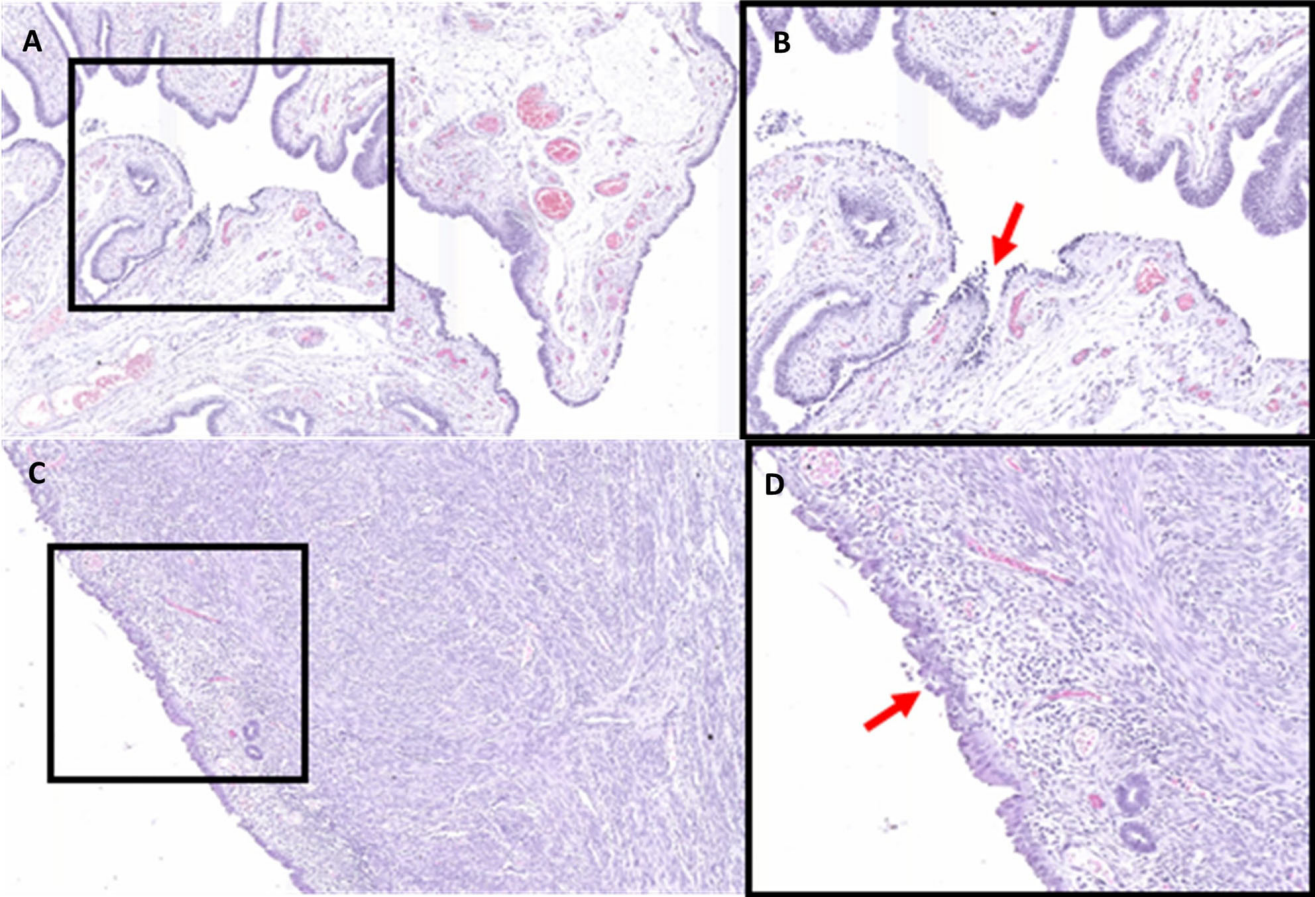
Hematoxylin and Eosin stained fallopian tube **(A, B)** and endometrium tissue **(C, D)** at 6× **(A, C)** and 12× **(B, D)** magnification. Areas of STIC **(B)** and EIC **(D)** are indicated by the red arrows.

Upon analysis of the H&E stained tissue sections, atypical intraepithelial proliferation involving a small population of cells in the endometrium were identified. These changes were consistent with early stages of EIC (**Table 1**). Similarly, atypical changes involving a small population of cells in the fimbriated tube (**Table 1**), consistent with early stages of STIC, were detected and both EIC and STIC are represented in **Figure 1**. Immunoperoxidase staining for p53 and MIB1 revealed atypical intraepithelial epithelial proliferations in both the fimbriated tube and endometrial lining, confirming the presence of STIC and EIC (data not shown).

### Proteomic Comparison of Healthy Epithelia to STIC and EIC

Regions of STIC, EIC, and adjacent healthy epithelium were laser microdissected (LMD) from sectioned tubal and endometrial specimens and analyzed by Nano-LC-MS/MS. In total, 453 proteins were detected across the 4 tissue types (369 proteins in the STIC, 110 proteins in healthy tubal epithelium, 428 proteins in EIC, and 162 proteins in healthy endometrial epithelium (**Supplementary Table 1**).

**Figure 2:**
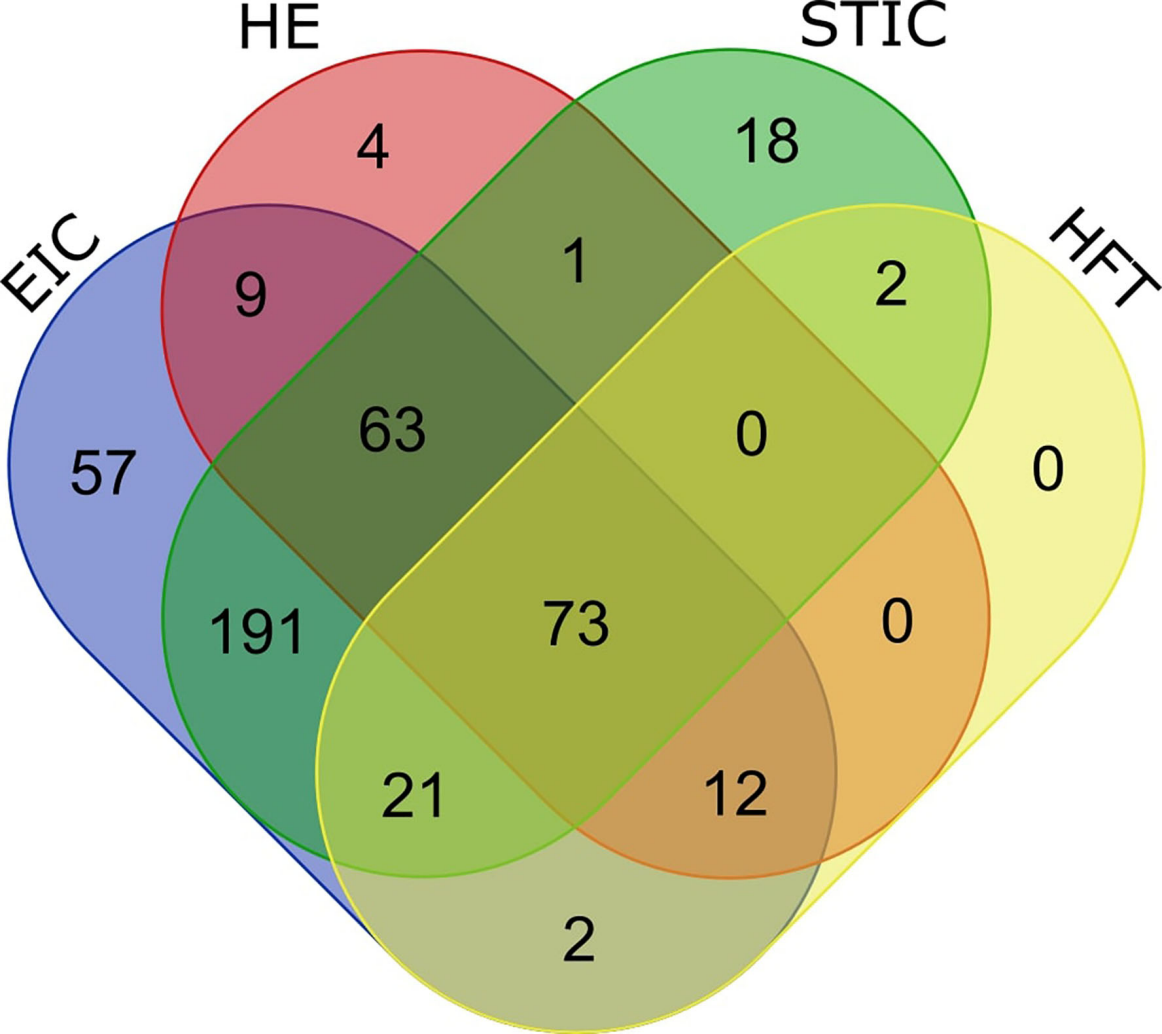
Venn diagram describing overlapping protein identifications in endometrial intraepithelial carcinoma (EIC), healthy endometrium (HE), serous tubal intraepithelial carcinoma (STIC) and healthy fallopian tube (HFT). A significant overlap of (348 proteins) is observed between EIC and STIC (diagram generated at http://bioinformatics.psb.ugent.be/webtools/Venn/).

When comparing the numbers of identified proteins, the greatest overlap occurred between STIC and EIC with 348 identical proteins identified in both samples. Across all tissue types 73 proteins were detectable, with 96 identical proteins identified in both the STIC and healthy tubal tissue, 157 identical proteins detected in both the EIC and healthy endometrial tissue, and 85 identical proteins were detected in both healthy tissues (**Figure 2**).

### Proteins Relevant to STIC, EIC, and the Development of Gynecological Cancer

In analyzing potential links between STIC and EIC cells and their respective invasive carcinomas, two of the identified proteins were of particular interest based on their involvement in gynecological cancer development: Epithelial cell adhesion molecule (EPCAM) and Calcyphosin (CAPS) (47–49). EPCAM was identified in both the STIC and EIC tissue samples but was not detected in either of the healthy tissues while CAPS was only detected in the healthy tube and in STIC.

### Expression of EPCAM and CAPS in Early Stage Serous Ovarian Carcinomas Compared to Healthy Peritoneal Tissue

To evaluate the expression of *EPCAM* and *CAPS* in early stage I serous ovarian carcinoma tissues, oligonucleotide microarray data was considered from the GEO data set GSE12470 (44). We chose to investigate the expression of these genes in early stage ovarian cancer as this is expected to be the stage following STIC in the development of serous ovarian cancer. STIC lesions were not deemed an appropriate comparison samples as they are often taken in the context of metastatic disease and potentially represent metastatic implants, and therefore more developed cancer, rather than true precursor lesions.

**Figure 3:**
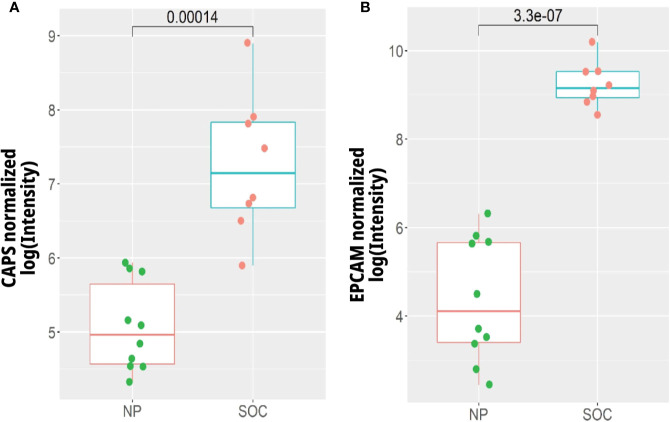
Gene expression of **(A)** CAPS and **(B)** EPCAM in early stage I serous ovarian cancer tissues (SOC) (n = 8) compared to normal peritoneum (NP) (n = 10). Expression levels were extracted from the data of Yoshihara et al. (44) (GEO Accession GSE12470) *via* the R package CuratedOvarianData (http://www.ncbi.nlm.nih.gov/geo/).

The gene expression levels were analyzed in 10 healthy peritoneal control tissues compared to 8 Stage I serous EOC tissues. The median expression levels of both *CAPS* and *EPCAM* were found to be significantly increased in the Stage I serous OC tissues compared to healthy peritoneal controls (p = 0.00014 and p = 3.3 × 10^−7^, respectively) (**Figure 3**).

Healthy OSE is infrequently available for research purposes given healthy ovaries are rarely removed during any type of medical procedure. Healthy peritoneum is an effective control because the lining of the ovaries is comprised of a single-cell mesothelial layer of poorly differentiated epithelium derived from the coelomic epithelium and extended to the serosa peritoneal cavity (50). In addition, it has been reported that peritoneal mesothelium and OSE are structurally very similar (51) and are both negative for *EPCAM* and *CAPS* expression (52, 53).

### Expression of EPCAM and CAPS in Early Stage Endometrial Carcinoma Compared to Healthy Endometrial Tissue

**Figure 4:**
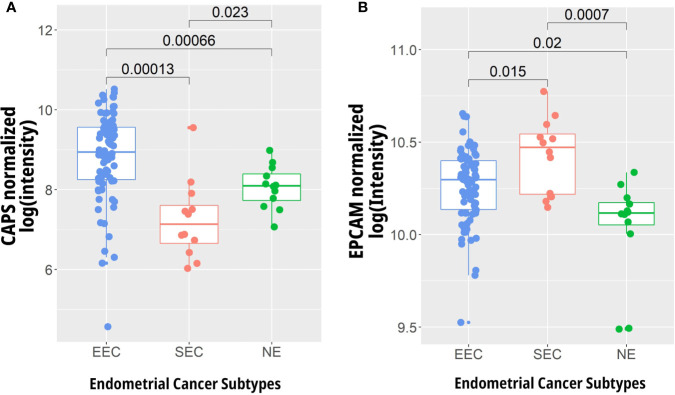
Gene expression analysis of **(A)** CAPS and **(B)** EPCAM in early stage endometrioid (EEC) (n = 79) and serous (SEC) endometrial carcinoma (n = 12) compared to normal endometrium (NE) (n = 12). Expression levels were extracted from the data of Days et al. (46) (GEO Accession GDS4589, http://www.ncbi.nlm.nih.gov/geo/) using R.

To assess the expression of *EPCAM* and *CAPS* in early stage endometrial cancer, oligonucleotide microarray data was considered from the GEO data set GDS4589. The gene expression levels were analyzed from 79 endometrioid and 12 serous papillary endometrial carcinomas as well as 12 healthy controls. The median expression of CAPS was increased significantly in EEC (p = 0.00066), while it was significantly decreased in SEC (p = 0.023) compared to healthy endometrium. The median expression of EPCAM was significantly increased in EEC (p = 0.02) and further increased in SEC (p = 0.0007) compared to healthy controls (**Figure 4**).

### Expression of Proteins Identified Exclusively in the STIC or EIC Across Ovarian Cancer Subtypes

Proteins detected exclusively in either the STIC or EIC tissues were analyzed to determine if their expression is specific to certain gynecological tissues. Marker proteins expressed exclusively by the precancerous cells of either the tube or endometrium, which are also expressed by ovarian carcinomas, may aid in determining the tissue specific origin of HGSOC. Eighteen proteins were detected exclusively in the STIC tissue and 57 proteins in the EIC tissue. Of these proteins a small number appear to be specific to certain gynecological tissues according to Protein Atlas; 2 identified from STIC (MIEAP, TPPP3), and 3 identified from EIC (ERO1A, DHSO, CSPG2/*VCAN*). These proteins and their tissue specificities across all gynecological tissues are listed in **Table 2**. The remaining proteins appeared to be more homogenously expressed and hence were not analyzed further.

**Table 2:** Proteins identified exclusively in the STIC or EIC that appear to be specific to certain gynecological tissues.

Protein	Detected In	Protein abundance levels in healthy tissues *	Protein abundance in ovarian cancer *	Protein abundance in endometrial cancer *
Mitochondrial eating protein (MIEAP), *SPATA18* ^#^	STIC	High in fallopian tube, low in endometrium and breast. Not expressed in the ovary.	Low	Low
Tubulin polymerization-promoting protein member 3 (TPPP3), *TPPP3*	STIC	Medium in glandular cells of the endometrium and cervix. Highly enriched in tube, negative in ovaries.	Medium	Medium to high
Endoplasmic reticulum oxidoreductin1-like protein alpha (ERO1A), *ERO1A* ^#^	EIC	High in cervix and medium in endometrium. No expression in tube or ovary.	Low	Low
Sorbitol dehydrogenase (DHSO), *SORD*	EIC	High in endometrium, cervix/uterinus, and low in breast. Negative in tube and ovaries.	Low	Medium
Versican core protein (CSPG2), *VCAN*	EIC	High in placenta. Medium in cervix/uterus, and endometrium. Low in tube and ovaries.	Low to medium	Low to Medium

*According to Protein Atlas (http://www.proteinatlas.org/).

^#^Not present in the Hendrix et al. data set. (45).

**Figure 5:**
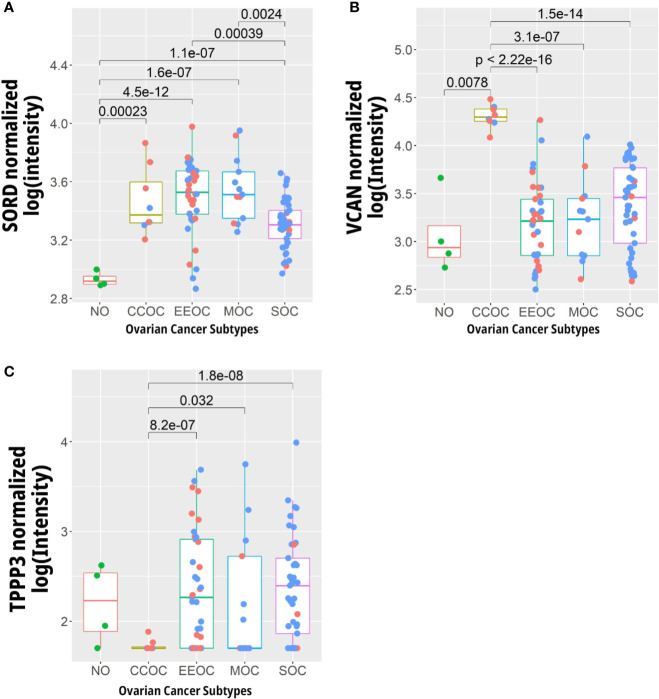
Expression levels of genes whose protein abundance is specific to certain to gynaecological tissues. **(A)** SORD, **(B)** TPP3, and **(C)** VCAN in 4 normal ovarian (NO), 8 clear cell (CCOC), 37 endometrial (EEOC), 13 mucinous (MOC), and 41 serous (SOC) ovarian carcinomas. Blue dots represent data points from late stage patients while the red represent early stage. Data gathered from Hendrix et al. (45) (GEO Accession GSE6008, http://www.ncbi.nlm.nih.gov/geo/).

The gene expression levels corresponding to the proteins listed in **Table 2** were compared using oligonucleotide microarray data from the GEO data set GSE6008 (**Figure 5**). For the gene *TPPP3* there was reduced expression in ovarian clear cell carcinoma compared to healthy tissue and other ovarian cancer subtypes. *SORD* showed significantly lower expression levels in the normal control tissues, and to a smaller extent, in serous ovarian carcinoma compared to the other subtypes. The high expression of *SORD*, whose related protein was detected only in EIC in our data set, in non-SOC was unexpected as the protein atlas reports low expression of *SORD* in ovarian cancer. CSPG2/*VCAN* expression, which was detected at the protein level in EIC only, was increased in CCOC when compared to the normal tissue and other EOC subtypes (**Figure 5**).

### Expression of Proteins Identified Exclusively in STIC or EIC Across Endometrial Cancer Subtypes

**Figure 6:**
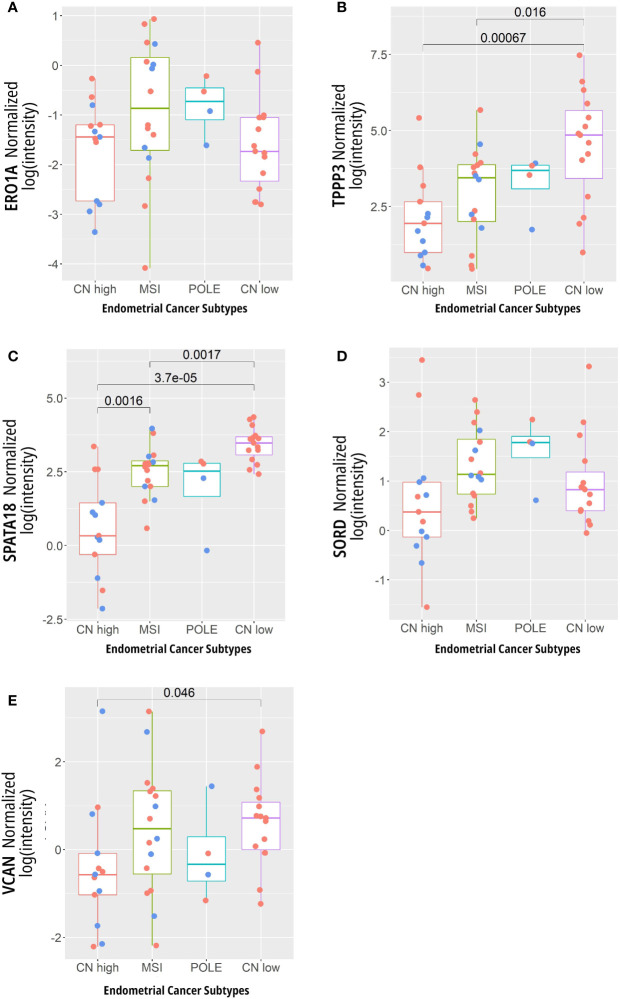
Expression levels of genes whose proteins are associated with specific gynecological tissues. **(A)** ERO1A, **(B)** TPPP3, **(C)** SPATA18, **(D)** SORD, and **(E)** VCAN in 13 copy number (CN) high, 16 micro satellite instability (MSI) hypermutated, 4 POLE ultra-mutated, and 15 CN low. Here, the blue dots represent data points from late stage patients while the red represent early stage cancers. Data from Kandolth et al. (4) (https://gdc.cancer.gov/node/875).

By investigating the gene expression corresponding to proteins in our data set which are enriched in specific gynecological tissues, we aimed to investigate the connection between preneoplastic lesions of the endometrium and tube with subtypes of endometrial cancer as defined by the TCGA. The expression levels of the genes corresponding to the proteins listed in **Table 1** were compared using oligonucleotide microarray data from Kandolth et al. (4) (https://gdc.cancer.gov/node/875) (**Figure 6**).

They were selected as their related proteins were found exclusively in a precancerous lesion and their abundance (according to protein atlas) was unique to specific gynecological tissue. However, most did not show different gene expression between the TCGA defined subtypes, except for *TPPP3* and *SPATA18*, which both showed decreased expression in the CN high (serous) subgroup compared to other subtypes (**Figure 6**). These proteins were detected exclusively in STIC in our analysis and their expression was seen to be decreased in SEC compared to healthy controls (**Supplementary Figure 2**).

### Proteins Associated With Metastasis Identified in STIC or EIC

The 34 identified proteins expressed exclusively in STIC and/or EIC which are known to be involved in metastasis or migration were listed in **Supplementary Table 2**. Most of these proteins are implicated in the promotion of metastasis, with the exceptions of Galectin-9 (54, 55), Mimecan (56, 57), and Catenin alpha-1 (58, 59) which have been implicated in the inhibition of metastasis.

## Discussion

The application of proteomic techniques, particularly that of mass spectrometry, hold the potential to provide a temporal snapshot of the molecular features within a given sample. Here, we provide what is, to the best of our knowledge, the first proteomic analyses of synchronous precursor lesions of serous endometrial and high-grade ovarian cancer. Through histological and molecular identification of precancerous lesions, followed by laser-capture microdissection and mass spectrometry analysis, we were able to identify over 450 proteins within the precancerous lesions and adjacent healthy tissue. The proteomic profiles of the precancerous lesions showed striking similarity (**Figure 1**) and shared a molecular profile indicative of metastatic transformation (**Supplementary Table 2**). To investigate the connection between these precancerous lesions and serous gynecological malignancies we investigated the gene expression of several proteins of interest in serous endometrial and ovarian cancer data sets (**Figures 3**–**6**).

EPCAM and CAPS were selected for further investigation based on their implication in the development of ovarian and endometrial cancer (47–49). Genomic analysis of their expression in Stage I SOC compared to peritoneal control tissue revealed significantly increased levels in the cancerous specimens (**Figure 3**). It is well recognized that EPCAM expression has a complex relationship to SOC development (60); however, it has not previously been identified in STIC or other gynecological precancerous lesions. This transmembrane adhesion molecule plays a role in migration and proliferation in wound healing (61) as well as the maintenance of pluripotency in stem cells (62).

The exact function of CAPS is unknown, but it is a suggested target of cAMP-dependent protein kinase and has been implicated in the cAMP and calcium-phosphatidylinositol signaling cascades (63). According to protein atlas, CAPS has high expression in the fallopian tube but is not expressed in healthy OSE (http://www.proteinatlas.org/ENSG00000152611-CAPSL/tissue) which is in agreement with our identification of this protein in both the healthy fallopian tissue and STIC. Interestingly, *CAPS* gene expression was seen to be increased in SOC compared to control tissue (**Figure 3**). Furthermore, *EPCAM* is not expressed in healthy ovarian surface epithelium (OSE) (53) but is frequently up regulated in ovarian cancer (60). The single case study presented here is insufficient to draw broader conclusions about potential markers of tissues of origin, but we believe that expression of EPCAM and CAPS merit further investigation in a larger cohort.

To further investigate proteins which may act as markers of tissue of origin in SEC or SOC, we investigated proteins identified exclusively in either the STIC or EIC which were unique to specific gynecological tissues according to protein atlas. CSGP/*VCAN* was identified exclusively in EIC, was seen to have increased expression in COCC compared to other ovarian cancer cell types (**Figure 5**) and had increased expression in SEC compared to healthy control tissue (**Supplementary Figure 2**). This protein has been previously seen to be increased in COCC (64, 65) and represents a potential link between EIC and COCC. COCC has been suggested to arise from endometriosis lesions (66) potentially representing a pathway for endometrial origin of COCC.

A previous mass spectrometry-based analysis of ovarian cancer precursor lesions was performed by Levenon et al. (67) and investigated *ex vivo* culture derived cells from fallopian tube fimbria (67). Their proteomic analysis identified 11 different ovarian cancer biomarkers present in this *ex vivo* model. A larger study by M. Eckart et al. (68) investigated both tumor and stroma tissue from STIC, invasive fallopian tube lesions, invasive ovarian lesions and omental metastasis (68). In addition to identifying N-methyltransferase (NNMT) as a metabolic regulator of cancer associated fibroblasts, they also showed that the molecular profiles of primary cancers and metastatic implants were remarkably similar within the same patient while the microenvironment showed site specific differences. The STIC lesion they investigated showed lower expression compared to normal tube in 4 of our proteins of interest (CAPS, ERO1A, TPPP3, and SPATA18) and similar expression in 2 (EPCAM and SORD). Only VCAN showed marginally increased expression in STIC compared to normal fallopian tube epithelium in this analysis. The authors’ focus on stromal tissue, a lack of normal epithelial ovarian or omental controls and the potential that STIC represents metastatic lesions from a primary ovarian tumor limits further comparison to this data set.

The traditional understanding of cancer development is that it acquires metastatic capacities over time within the primary lesion (69). However, the identification of STIC as the origin site of HGSOC implicates that these pre-invasive lesions can leave their site of origin and establish themselves in distant locations. In addition, EIC is often identified with extrauterine spread (18, 19) which can take the form of EIC like growths on the ovaries, peritoneum and fallopian tube in the absence of obvious disease in these locations (16, 18). Together, this suggests that these premalignant lesions possess some migratory or metastatic ability facilitating their translocation to distant sites within the gynecological system. Here, we identified numerous metastasis and migration related proteins in precancerous lesions in the absence of malignant disease (**Supplementary Table 2**).

A major limitation of this study is that the data is derived from a single patient case study which makes generalized interpretation of biological implication of this data difficult. Furthermore, only a modest number of proteins was identified from these small tissue regions. As highly abundant proteins are identified preferentially in mass spectrometry analysis (70), the low number of proteins identified potentially masks differences in lower abundance proteins. The analysis of a larger cohort, coupled with the utilization of advanced sample preparation and mass spectrometry techniques to improve proteome coverage, holds the potential to build upon the data presented here and paint a clearer picture of the molecular landscape of precancerous lesions of serous cancers of the ovary and endometrium.

## Conclusion

Here, we present the first proteomic investigation of precancerous lesions of the gynecological tract in a patient without advanced gynecological malignancy. Interpretation of the data is limited by the single case study and the modest number of proteins identified; however, we provide a foundation for further analysis of the molecular links between precancerous lesions and subsequent caner. In addition, we identified several metastasis-related proteins in precancerous tissues. The understanding that precancerous lesions of the female genital tract potentially possess metastatic potential raises many questions about when, where, and how these cancers develop. Though the early steps are not well understood, further proteomic analyses of gynecological precancerous lesions hold the potential to unravel the early temporal and molecular events underlying the development of these malignancies which, in turn, holds the potential to improve detection and treatment.

## Data Availability Statement

Publicly available datasets were analyzed in this study. These can be found in the NCBI Gene Expression Omnibus. The mass spectrometry proteomics data have been deposited to the ProteomeXchange Consortium via the PRIDE partner repository (71) with the data set identifier PXD018538.

## Ethics Statement

The studies involving human participants were reviewed and approved by Research Ethics Committee of the Royal Adelaide Hospital. The patients/participants provided their written informed consent to participate in this study.

## Author Contributions

Design of the work: MO, PH, GA, and MA. Acquisition, analysis, and interpretation of data: all authors. Drafting the work or revising it critically: all authors. All authors contributed to the article and approved the submitted version.

## Funding

PH gratefully acknowledges the support of the National Collaborative Research Infrastructure Strategy (NCRIS) Bioplatforms Australia Node for Tissue Imaging Mass Spectrometry.

## Conflict of Interest

Author FW was employed by the company Clinpath.

The remaining authors declare that the research was conducted in the absence of any commercial or financial relationships that could be construed as a potential conflict of interest.
