# Supplementary material for: Proteomic Analysis of Pre-Invasive Serous Lesions of the Endometrium and Fallopian Tube Reveals Their Metastatic Potential
Source: Front Oncol. 2020 Dec 15;10:523989. doi: 10.3389/fonc.2020.523989 (PMC7771701; doi:10.3389/fonc.2020.523989)
Supplement: Supplementary file 2 [file DataSheet_2.docx]

**Supplementary Table 2**. Proteins associated with metastasis, identified exclusively in the STIC and/ or EIC

| **Protein** | **Detected In** | **Biological Significance** |
| --- | --- | --- |
| Adenylyl cyclase-associated protein 1 (CAP1) | STIC and EIC | Associated with metastasis in lung and breast cancers (Tan, Song et al. 2013, Zhang and Zhou 2016) |
| Poly r(C) binding protein-1 (PCBP1) | STIC and EIC | Integral to the maintenance of stem-cell-like prostate cancer cells (Chen, Cai et al. 2015) |
| Lamina associated polypeptide 2 (LAP2A) | STIC and EIC | Increased in abundance in digestive tract cancers and has been linked to metastasis via the regulation of cell motility (Kim, Hwang et al. 2012) |
| Protein disulfide isomerase A (PDIA) | STIC and EIC | Linked to metastasis in hepatocellular carcinoma (Chen, Sun et al. 2008) |
| Transketolase (TKT) | STIC and EIC | Identified as a promoter of ovarian cancer cell proliferation and in up-regulated in peritoneal metastases (Ricciardelli, Lokman et al. 2015) |
| Annexin A11 (ANX11) | STIC and EIC | Promotes tumourigenesis, metastasis, and chemosensitivity in hepatocarcinoma (Liu, Wang et al. 2015, Liu, Guo et al. 2016) |
| Cytoskeleton-associated protein 4 (CKAP4) | STIC and EIC | Expression associated with metastasis in intrahepatic cholangiocellular carcinoma (Li, Dong et al. 2013) |
| Myoferlin (MYOF) | STIC and EIC | Influences the invasive capacity of breast cancer cells (Volakis, Li et al. 2014) |
| Glutamate dehydrogenase 2 (DHE3) | STIC and EIC | Over-expression of glutamate dehydrogenase promotes cell proliferation, migration and invasion *in vivo* in colorectal cancer (Liu, Zhu et al. 2015) |
| Transcription factor A (TFAM) | STIC and EIC | Shown potential in predicting the clinical outcome of metastatic colorectal cancer patients treated with chemotherapies (Yoshida, Hasegawa et al. 2011) |
| Elongation factor 1-delta (EF1D) | STIC and EIC | Increased expression correlates with lymph node metastasis in oesophageal cancer (Ogawa, Utsunomiya et al. 2004) |
| Agrin (AGRIN) | STIC and EIC | Associated with cell migration and adhesion in oral squamous cell carcinoma (Kawahara, Granato et al. 2014) |
| Moesin (MOES) | STIC and EIC | Involved in the metastatic spread of several tumours (Lallemand and Arpin 2010) |
| Kinectin (KTN1) | STIC and EIC | Supports the development of focal adhesions (Zhang, Tee et al. 2010) |
| Biliverdin reductase A (BIEA) | STIC and EIC | Biliverdin reductase shown to promote epithelial-mesenchymal transition in breast cancer (Zhang, Song et al. 2016) |
| Chloride intracellular channel protein 1 (CLIC1) | STIC and EIC | Regulates invasion and migration in colon cancer (Wang, Zeng et al. 2014), and is correlates with poorer prognosis in gliomas (Wang, He et al. 2012) |
| Zyxin (ZYX) | STIC and EIC | Involved in the reorganisation of acting fibres to enhance cell motility (Mori, Nakagami et al. 2009) |
| Claudin-3 (CLD3) | STIC and EIC | Over expression linked to enhanced cellular motility in breast cancer cell lines (Todd, Petty et al. 2015) |
| Mimecan (MIME) | STIC and EIC | Expression decreased metastatic capacity of mouse hepatocarcinoma cells (Cui, Song et al. 2008, Cui, Tang et al. 2009) |
| Annexin A4 (ANXA4) | STIC and EIC | Associated with poor prognosis and metastasis of hepatocellular carcinoma (Chen, Chen et al. 2016) |
| Far upstream element binding protein 1 (FUBP1) | STIC and EIC | Promotes carcinoma progression and migration (Zhang and Chen 2013, Liu, Hu et al. 2015) |
| Galectin-9 (LEG9) | STIC and EIC | Shown to suppress tumour metastasis (Nobumoto, Nagahara et al. 2008, Fujihara, Mori et al. 2013) |
| Rho GDP-dissociation inhibitor 1 (GDIR1) | STIC and EIC | Associated with metastasis in colorectal cancer (Yamashita, Okamura et al. 2012) |
| PDZ and LIM domain protein 1 (PDLI1) | STIC and EIC | Promotes migration, invasion and metastasis through interaction with α-actinin in breast cancer (Liu, Zhan et al. 2015) |
| ADP-ribosylation factor-like 4C (ARF4) | STIC and EIC | Associated with suppression of metastasis in ovarian cancer (Su, Katsaros et al. 2015) |
| Glucose-6-phosphate dehydrogenase (G6PD) | STIC and EIC | Implicated in the survival, proliferation, and metastasis of cancer cells (Zhang, Zhang et al. 2014) |
| Aldehyde dehydrogenase (ALDH2) | STIC and EIC | Mediator of metastasis in several solid tumours (Rodriguez-Torres and Allan 2016) |
| Cathepsin B (CATB) | STIC and EIC | Shown to mediate tumour metastasis (Tan, Peng et al. 2013) |
| Dimethylarginine dimethylaminohydrolase 2 (DDAH2) | STIC and EIC | Associated with invasiveness and angiogenesis in lung adenocarcinoma (Shiozawa, Iyama et al. 2016) |
| Ceruloplasmin (CERU) | STIC and EIC | Plasma levels elevated in metastatic breast cancer patients (Schapira and Schapira 1983) |
| Microtubule associated protein-4 (MAP4) | STIC and EIC | Regulator of invasion and migration in esophageal squamous cell carcinoma (Jiang, Shang et al. 2016) |
| Catenin alpha-1 (CTNA1) | STIC and EIC | Suppressor of metastasis (Beavon 2000, Yoshida, Kimura et al. 2001) |
| Tubulin polymerisation promoting proteins (TPPP3) | STIC | Knock down suppresses tumour proliferation and metastasis in lung cancer (Li, Xu et al. 2016) (Zhou, Li et al. 2010) |
| Membrane-associated progesterone receptor component 1 (PGRC1) | EIC | Promotes tumour growth by binding to and stabilising EGFR at the plasma membrane (Ahmed, Rohe et al. 2010). Found to promote tumor cell viability during chemotherapeutic stress in endometrial cancer (Friel, Zhang et al. 2015). |
| Myristoylated alanine-rich C-kinase substrate (MARCS) | EIC | Enhances the migration and metastasis of lung and biliary carcinoma (Techasen, Loilome et al. 2010, Chen, Statt et al. 2014, Chen, Thai et al. 2014). |
| Prolactin-inducible protein (PIP) | EIC | Suggested marker of breast and prostate cancer metastasis (Clark, Snell et al. 1999, Hassan, Waheed et al. 2009, Guetschow, Black et al. 2012). |
| Superoxide dismutase [Mn] (SODM) | EIC | Involved in redox-responsive signaling events that drive invasion, migration, and tumor cell survival (Hempel, Carrico et al. 2011). |
| F-actin-capping protein subunit alpha-1 (CAZA1) | EIC | Inhibition of CapG protein reduces breast cancer metastasis (Van Impe, Bethuyne et al. 2013). |
| Retinal dehydrogenase 1 (AL1A1) | EIC | Implicated in breast cancer metastasis (Charafe-Jauffret, Ginestier et al. 2010, Dong, Bi et al. 2013). |
| Thioredoxin domain-containing protein 5 (TXND5) | EIC | Highly expressed in metastatic gastric adenocarcinoma (Wu, Zhang et al. 2015). |
| Suprabasin (SBSN) | EIC | Involved in salivary adenoid carcinoma metastasis (Shao, Tan et al. 2012) and in oesophageal squamous cell carcinoma proliferation and tumourigenesis (Zhu, Wu et al. 2016). |
| Carnitine palmitoyltransferase 1A (CPT1A) | EIC | Promotes cell motility in alveolar rhabdomyosarcoma cells (Liu, Wang et al. 2012). |
| Tenascin (TENA) | EIC | Reported to play an important role in the metastasis of several cancers (Lowy and Oskarsson 2015). |
| Palladin (PALLD) | EIC | Regulates actin cytoskeleton organisation and cell adhesion (Najm and El-Sibai 2014), promotes pancreatic and breast cancer invasion (Goicoechea, Bednarski et al. 2009, Goicoechea, Garcia-Mata et al. 2014). |
| Tight junction protein ZO-1 (ZO1) | EIC | Differential expression in metastatic pancreatic and liver cancer (Kleeff, Shi et al. 2001, Orban, Szabo et al. 2008). |

Ahmed, I. S., H. J. Rohe, K. E. Twist and R. J. Craven (2010). "Pgrmc1 (progesterone receptor membrane component 1) associates with epidermal growth factor receptor and regulates erlotinib sensitivity." J Biol Chem **285**(32): 24775-24782.

Beavon, I. R. (2000). "The E-cadherin-catenin complex in tumour metastasis: structure, function and regulation." Eur J Cancer **36**(13 Spec No): 1607-1620.

Charafe-Jauffret, E., C. Ginestier, F. Iovino, C. Tarpin, M. Diebel, B. Esterni, G. Houvenaeghel, J. M. Extra, F. Bertucci, J. Jacquemier, L. Xerri, G. Dontu, G. Stassi, Y. Xiao, S. H. Barsky, D. Birnbaum, P. Viens and M. S. Wicha (2010). "Aldehyde dehydrogenase 1-positive cancer stem cells mediate metastasis and poor clinical outcome in inflammatory breast cancer." Clin Cancer Res **16**(1): 45-55.

Chen, C. H., S. Statt, C. L. Chiu, P. Thai, M. Arif, K. B. Adler and R. Wu (2014). "Targeting myristoylated alanine-rich C kinase substrate phosphorylation site domain in lung cancer. Mechanisms and therapeutic implications." Am J Respir Crit Care Med **190**(10): 1127-1138.

Chen, C. H., P. Thai, K. Yoneda, K. B. Adler, P. C. Yang and R. Wu (2014). "A peptide that inhibits function of Myristoylated Alanine-Rich C Kinase Substrate (MARCKS) reduces lung cancer metastasis." Oncogene **33**(28): 3696-3706.

Chen, N., W. Sun, X. Deng, Y. Hao, X. Chen, B. Xing, W. Jia, J. Ma, H. Wei, Y. Zhu, X. Qian, Y. Jiang and F. He (2008). "Quantitative proteome analysis of HCC cell lines with different metastatic potentials by SILAC." Proteomics **8**(23-24): 5108-5118.

Chen, Q., Z. K. Cai, Y. B. Chen, M. Gu, D. C. Zheng, J. Zhou and Z. Wang (2015). "Poly r(C) binding protein-1 is central to maintenance of cancer stem cells in prostate cancer cells." Cell Physiol Biochem **35**(3): 1052-1061.

Chen, W., L. Chen, Z. Cai, D. Liang, B. Zhao, Y. Zeng, X. Liu and J. Liu (2016). "Overexpression of annexin A4 indicates poor prognosis and promotes tumor metastasis of hepatocellular carcinoma." Tumour Biol.

Clark, J. W., L. Snell, R. P. C. Shiu, F. W. Orr, N. Maitre, C. P. H. Vary, D. J. Cole and P. H. Watson (1999). "The potential role for prolactin-inducible protein (PIP) as a marker of human breast cancer micrometastasis." British Journal of Cancer **81**(6): 1002-1008.

Cui, X.-N., J.-W. Tang, B. Song, B. Wang, S.-Y. Chen and L. Hou (2009). "High expression of osteoglycin decreases gelatinase activity of murine hepatocarcinoma Hca-F cells." World Journal of Gastroenterology : WJG **15**(48): 6117-6122.

Cui, X., B. Song, L. Hou, Z. Wei and J. Tang (2008). "High expression of osteoglycin decreases the metastatic capability of mouse hepatocarcinoma Hca-F cells to lymph nodes." Acta Biochim Biophys Sin (Shanghai) **40**(4): 349-355.

Dong, Y., L. R. Bi, N. Xu, H. M. Yang, H. T. Zhang, Y. Ding, A. P. Shi and Z. M. Fan (2013). "The expression of aldehyde dehydrogenase 1 in invasive primary breast tumors and axillary lymph node metastases is associated with poor clinical prognosis." Pathol Res Pract **209**(9): 555-561.

Friel, A. M., L. Zhang, C. A. Pru, N. C. Clark, M. L. McCallum, L. J. Blok, T. Shioda, J. J. Peluso, B. R. Rueda and J. K. Pru (2015). "Progesterone receptor membrane component 1 deficiency attenuates growth while promoting chemosensitivity of human endometrial xenograft tumors." Cancer letters **356**(2 0 0): 434-442.

Fujihara, S., H. Mori, H. Kobara, K. Rafiq, T. Niki, M. Hirashima and T. Masaki (2013). "Galectin-9 in cancer therapy." Recent Pat Endocr Metab Immune Drug Discov **7**(2): 130-137.

Goicoechea, S. M., B. Bednarski, R. Garcia-Mata, H. Prentice-Dunn, H. J. Kim and C. A. Otey (2009). "Palladin contributes to invasive motility in human breast cancer cells." Oncogene **28**(4): 587-598.

Goicoechea, S. M., R. Garcia-Mata, J. Staub, A. Valdivia, L. Sharek, C. G. McCulloch, R. F. Hwang, R. Urrutia, J. J. Yeh, H. J. Kim and C. A. Otey (2014). "Palladin promotes invasion of pancreatic cancer cells by enhancing invadopodia formation in cancer-associated fibroblasts." Oncogene **33**(10): 1265-1273.

Guetschow, E. D., W. Black, C. M. Walsh and J. R. Furchak (2012). "Detection of prolactin inducible protein mRNA, a biomarker for breast cancer metastasis, using a molecular beacon-based assay." Anal Bioanal Chem **404**(2): 399-406.

Hassan, M. I., A. Waheed, S. Yadav, T. P. Singh and F. Ahmad (2009). "Prolactin inducible protein in cancer, fertility and immunoregulation: structure, function and its clinical implications." Cell Mol Life Sci **66**(3): 447-459.

Hempel, N., P. M. Carrico and J. A. Melendez (2011). "Manganese superoxide dismutase (Sod2) and redox-control of signaling events that drive metastasis." Anti-cancer agents in medicinal chemistry **11**(2): 191-201.

Jiang, Y. Y., L. Shang, Z. Z. Shi, T. T. Zhang, S. Ma, C. C. Lu, Y. Zhang, J. J. Hao, C. Shi, F. Shi, X. Xu, Y. Cai, X. M. Jia, Q. M. Zhan and M. R. Wang (2016). "Microtubule-associated protein 4 is an important regulator of cell invasion/migration and a potential therapeutic target in esophageal squamous cell carcinoma." Oncogene.

Kawahara, R., D. C. Granato, C. M. Carnielli, N. K. Cervigne, C. E. Oliveria, C. Rivera, S. Yokoo, F. P. Fonseca, M. Lopes, A. R. Santos-Silva, E. Graner, R. D. Coletta and A. F. Paes Leme (2014). "Agrin and perlecan mediate tumorigenic processes in oral squamous cell carcinoma." PLoS One **9**(12): e115004.

Kim, H.-J., S.-H. Hwang, M.-E. Han, S. Baek, H.-E. Sim, S. Yoon, S.-Y. Baek, B.-S. Kim, J.-H. Kim, S.-Y. Kim and S.-O. Oh (2012). "LAP2 Is Widely Overexpressed in Diverse Digestive Tract Cancers and Regulates Motility of Cancer Cells." PLoS ONE **7**(6): e39482.

Kleeff, J., X. Shi, H. P. Bode, K. Hoover, S. Shrikhande, P. J. Bryant, M. Korc, M. W. Buchler and H. Friess (2001). "Altered expression and localization of the tight junction protein ZO-1 in primary and metastatic pancreatic cancer." Pancreas **23**(3): 259-265.

Lallemand, D. and M. Arpin (2010). "Moesin/ezrin: a specific role in cell metastasis?" Pigment Cell Melanoma Res **23**(1): 6-7.

Li, M. H., L. W. Dong, S. X. Li, G. S. Tang, Y. F. Pan, J. Zhang, H. Wang, H. B. Zhou, Y. X. Tan, H. P. Hu and H. Y. Wang (2013). "Expression of cytoskeleton-associated protein 4 is related to lymphatic metastasis and indicates prognosis of intrahepatic cholangiocarcinoma patients after surgery resection." Cancer Lett **337**(2): 248-253.

Li, Y., Y. Xu, K. Ye, N. Wu, J. Li, N. Liu, M. He, B. Lu, W. Zhou and R. Hu (2016). "Knockdown of Tubulin Polymerization Promoting Protein Family Member 3 Suppresses Proliferation and Induces Apoptosis in Non-Small-Cell Lung Cancer." Journal of Cancer **7**(10): 1189-1196.

Liu, G., J. Zhu, M. Yu, C. Cai, Y. Zhou, M. Yu, Z. Fu, Y. Gong, B. Yang, Y. Li, Q. Zhou, Q. Lin, H. Ye, L. Ye, X. Zhao, Z. Li, R. Chen, F. Han, C. Tang and B. Zeng (2015). "Glutamate dehydrogenase is a novel prognostic marker and predicts metastases in colorectal cancer patients." J Transl Med **13**: 144.

Liu, L., Y. D. Wang, J. Wu, J. Cui and T. Chen (2012). "Carnitine palmitoyltransferase 1A (CPT1A): a transcriptional target of PAX3-FKHR and mediates PAX3-FKHR-dependent motility in alveolar rhabdomyosarcoma cells." BMC Cancer **12**: 154.

Liu, S., C. Guo, J. Wang, B. Wang, H. Qi and M. Z. Sun (2016). "ANXA11 regulates the tumorigenesis, lymph node metastasis and 5-fluorouracil sensitivity of murine hepatocarcinoma Hca-P cells by targeting c-Jun." Oncotarget **7**(13): 16297-16310.

Liu, S., J. Wang, C. Guo, H. Qi and M. Z. Sun (2015). "Annexin A11 knockdown inhibits in vitro proliferation and enhances survival of Hca-F cell via Akt2/FoxO1 pathway and MMP-9 expression." Biomed Pharmacother **70**: 58-63.

Liu, Z., Y. Zhan, Y. Tu, K. Chen, Z. Liu and C. Wu (2015). "PDZ and LIM domain protein 1(PDLIM1)/CLP36 promotes breast cancer cell migration, invasion and metastasis through interaction with alpha-actinin." Oncogene **34**(10): 1300-1311.

Liu, Z. H., J. L. Hu, J. Z. Liang, A. J. Zhou, M. Z. Li, S. M. Yan, X. Zhang, S. Gao, L. Chen, Q. Zhong and M. S. Zeng (2015). "Far upstream element-binding protein 1 is a prognostic biomarker and promotes nasopharyngeal carcinoma progression." Cell Death Dis **6**: e1920.

Lowy, C. M. and T. Oskarsson (2015). "Tenascin C in metastasis: A view from the invasive front." Cell Adh Migr **9**(1-2): 112-124.

Mori, M., H. Nakagami, N. Koibuchi, K. Miura, Y. Takami, H. Koriyama, H. Hayashi, H. Sabe, N. Mochizuki, R. Morishita and Y. Kaneda (2009). "Zyxin Mediates Actin Fiber Reorganization in Epithelial–Mesenchymal Transition and Contributes to Endocardial Morphogenesis." Molecular Biology of the Cell **20**(13): 3115-3124.

Najm, P. and M. El-Sibai (2014). "Palladin regulation of the actin structures needed for cancer invasion." Cell Adhesion & Migration **8**(1): 29-35.

Nobumoto, A., K. Nagahara, S. Oomizu, S. Katoh, N. Nishi, K. Takeshita, T. Niki, A. Tominaga, A. Yamauchi and M. Hirashima (2008). "Galectin-9 suppresses tumor metastasis by blocking adhesion to endothelium and extracellular matrices." Glycobiology **18**(9): 735-744.

Ogawa, K., T. Utsunomiya, K. Mimori, Y. Tanaka, F. Tanaka, H. Inoue, S. Murayama and M. Mori (2004). "Clinical significance of elongation factor-1 delta mRNA expression in oesophageal carcinoma." Br J Cancer **91**(2): 282-286.

Orban, E., E. Szabo, G. Lotz, P. Kupcsulik, C. Paska, Z. Schaff and A. Kiss (2008). "Different expression of occludin and ZO-1 in primary and metastatic liver tumors." Pathol Oncol Res **14**(3): 299-306.

Ricciardelli, C., N. A. Lokman, S. Cheruvu, I. A. Tan, M. P. Ween, C. E. Pyragius, A. Ruszkiewicz, P. Hoffmann and M. K. Oehler (2015). "Transketolase is upregulated in metastatic peritoneal implants and promotes ovarian cancer cell proliferation." Clin Exp Metastasis **32**(5): 441-455.

Rodriguez-Torres, M. and A. L. Allan (2016). "Aldehyde dehydrogenase as a marker and functional mediator of metastasis in solid tumors." Clinical & Experimental Metastasis **33**: 97-113.

Schapira, D. V. and M. Schapira (1983). "Use of ceruloplasmin levels to monitor response to therapy and predict recurrence of breast cancer." Breast Cancer Res Treat **3**(2): 221-224.

Shao, C., M. Tan, J. A. Bishop, J. Liu, W. Bai, D. A. Gaykalova, T. Ogawa, A. R. Vikani, Y. Agrawal, R. J. Li, M. S. Kim, W. H. Westra, D. Sidransky, J. A. Califano and P. K. Ha (2012). "Suprabasin is hypomethylated and associated with metastasis in salivary adenoid cystic carcinoma." PLoS One **7**(11): e48582.

Shiozawa, T., S. Iyama, S. Toshima, A. Sakata, S. Usui, Y. Minami, Y. Sato, N. Hizawa and M. Noguchi (2016). "Dimethylarginine dimethylaminohydrolase 2 promotes tumor angiogenesis in lung adenocarcinoma." Virchows Archiv **468**: 179-190.

Su, D., D. Katsaros, S. Xu, H. Xu, Y. Gao, N. Biglia, J. Feng, L. Ying, P. Zhang, C. Benedetto and H. Yu (2015). "ADP-ribosylation factor-like 4C (ARL4C), a novel ovarian cancer metastasis suppressor, identified by integrated genomics." Am J Transl Res **7**(2): 242-256.

Tan, G.-J., Z.-K. Peng, J.-P. Lu and F.-Q. Tang (2013). "Cathepsins mediate tumor metastasis." World Journal of Biological Chemistry **4**(4): 91-101.

Tan, M., X. Song, G. Zhang, A. Peng, X. Li, M. Li, Y. Liu and C. Wang (2013). "Overexpression of adenylate cyclase-associated protein 1 is associated with metastasis of lung cancer." Oncol Rep **30**(4): 1639-1644.

Techasen, A., W. Loilome, N. Namwat, E. Takahashi, E. Sugihara, A. Puapairoj, M. Miwa, H. Saya and P. Yongvanit (2010). "Myristoylated alanine-rich C kinase substrate phosphorylation promotes cholangiocarcinoma cell migration and metastasis via the protein kinase C-dependent pathway." Cancer Sci **101**(3): 658-665.

Todd, M. C., H. M. Petty, J. M. King, B. N. Piana Marshall, R. A. Sheller and M. E. Cuevas (2015). "Overexpression and delocalization of claudin-3 protein in MCF-7 and MDA-MB-415 breast cancer cell lines." Oncology Letters **10**(1): 156-162.

Van Impe, K., J. Bethuyne, S. Cool, F. Impens, D. Ruano-Gallego, O. De Wever, B. Vanloo, M. Van Troys, K. Lambein, C. Boucherie, E. Martens, O. Zwaenepoel, G. Hassanzadeh-Ghassabeh, J. Vandekerckhove, K. Gevaert, L. A. Fernandez, N. N. Sanders and J. Gettemans (2013). "A nanobody targeting the F-actin capping protein CapG restrains breast cancer metastasis." Breast Cancer Res **15**(6): R116.

Volakis, L. I., R. Li, W. E. t. Ackerman, C. Mihai, M. Bechel, T. L. Summerfield, C. S. Ahn, H. M. Powell, R. Zielinski, T. J. Rosol, S. N. Ghadiali and D. A. Kniss (2014). "Loss of myoferlin redirects breast cancer cell motility towards collective migration." PLoS One **9**(2): e86110.

Wang, L., S. He, Y. Tu, P. Ji, J. Zong, J. Zhang, F. Feng, J. Zhao, Y. Zhang and G. Gao (2012). "Elevated expression of chloride intracellular channel 1 is correlated with poor prognosis in human gliomas." J Exp Clin Cancer Res **31**: 44.

Wang, P., Y. Zeng, T. Liu, C. Zhang, P.-W. Yu, Y.-X. Hao, H.-X. Luo and G. Liu (2014). "Chloride intracellular channel 1 regulates colon cancer cell migration and invasion through ROS/ERK pathway." World Journal of Gastroenterology : WJG **20**(8): 2071-2078.

Wu, Z., L. I. N. Zhang, N. A. N. Li, L. Sha and K. Zhang (2015). "An immunohistochemical study of thioredoxin domain-containing 5 expression in gastric adenocarcinoma." Oncology Letters **9**(3): 1154-1158.

Yamashita, T., T. Okamura, K. Nagano, S. Imai, Y. Abe, H. Nabeshi, T. Yoshikawa, Y. Yoshioka, H. Kamada, Y. Tsutsumi and S. Tsunoda (2012). "Rho GDP-dissociation inhibitor alpha is associated with cancer metastasis in colon and prostate cancer." Pharmazie **67**(3): 253-255.

Yoshida, R., N. Kimura, Y. Harada and N. Ohuchi (2001). "The loss of E-cadherin, alpha- and beta-catenin expression is associated with metastasis and poor prognosis in invasive breast cancer." Int J Oncol **18**(3): 513-520.

Yoshida, Y., J. Hasegawa, R. Nezu, Y. K. Kim, M. Hirota, K. Kawano, H. Izumi and K. Kohno (2011). "Clinical usefulness of mitochondrial transcription factor A expression as a predictive marker in colorectal cancer patients treated with FOLFOX." Cancer Sci **102**(3): 578-582.

Zhang, C., Z. Zhang, Y. Zhu and S. Qin (2014). "Glucose-6-phosphate dehydrogenase: a biomarker and potential therapeutic target for cancer." Anticancer Agents Med Chem **14**(2): 280-289.

Zhang, H. and G. L. Zhou (2016). "CAP1 (Cyclase-Associated Protein 1) Exerts Distinct Functions in the Proliferation and Metastatic Potential of Breast Cancer Cells Mediated by ERK." Sci Rep **6**: 25933.

Zhang, J. and Q. M. Chen (2013). "Far upstream element binding protein 1: a commander of transcription, translation and beyond." Oncogene **32**(24): 2907-2916.

Zhang, M., S. Song, Z. Yi, X. Zhao, L. Fu, L. Wang, C. Ma, M. Mao, Y. Xing and D. Zhu (2016). "Human biliverdin reductase promotes EMT through the ERK1/2 signal pathway in breast cancer." Eur J Pharmacol **788**: 45-53.

Zhang, X., Y. H. Tee, J. K. Heng, Y. Zhu, X. Hu, F. Margadant, C. Ballestrem, A. Bershadsky, G. Griffiths and H. Yu (2010). "Kinectin-mediated endoplasmic reticulum dynamics supports focal adhesion growth in the cellular lamella." Journal of Cell Science **123**(22): 3901-3912.

Zhou, W., J. Li, X. Wang and R. Hu (2010). "Stable knockdown of TPPP3 by RNA interference in Lewis lung carcinoma cell inhibits tumor growth and metastasis." Mol Cell Biochem **343**(1-2): 231-238.

Zhu, J., G. Wu, Q. Li, H. Gong, J. Song, L. Cao, S. Wu, L. Song and L. Jiang (2016). "Overexpression of Suprabasin is Associated with Proliferation and Tumorigenicity of Esophageal Squamous Cell Carcinoma." Scientific Reports **6**: 21549.
