# Supplementary figures and images for: Proteomic Analysis of Pre-Invasive Serous Lesions of the Endometrium and Fallopian Tube Reveals Their Metastatic Potential
Source: Front Oncol. 2020 Dec 15;10:523989. doi: 10.3389/fonc.2020.523989 (PMC7771701; doi:10.3389/fonc.2020.523989)

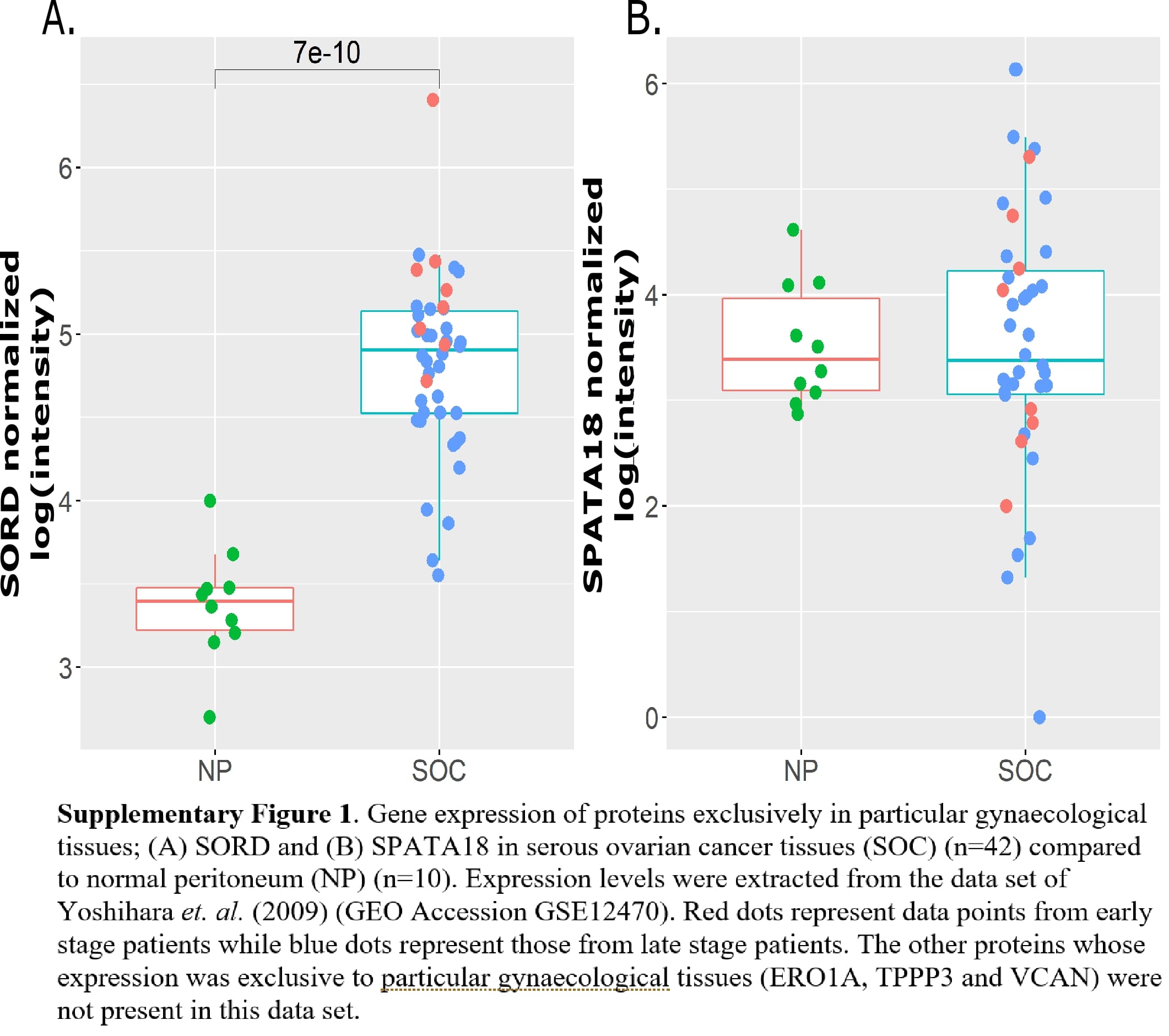

Supplement: Supplementary file 3 [file Image_1.jpeg]

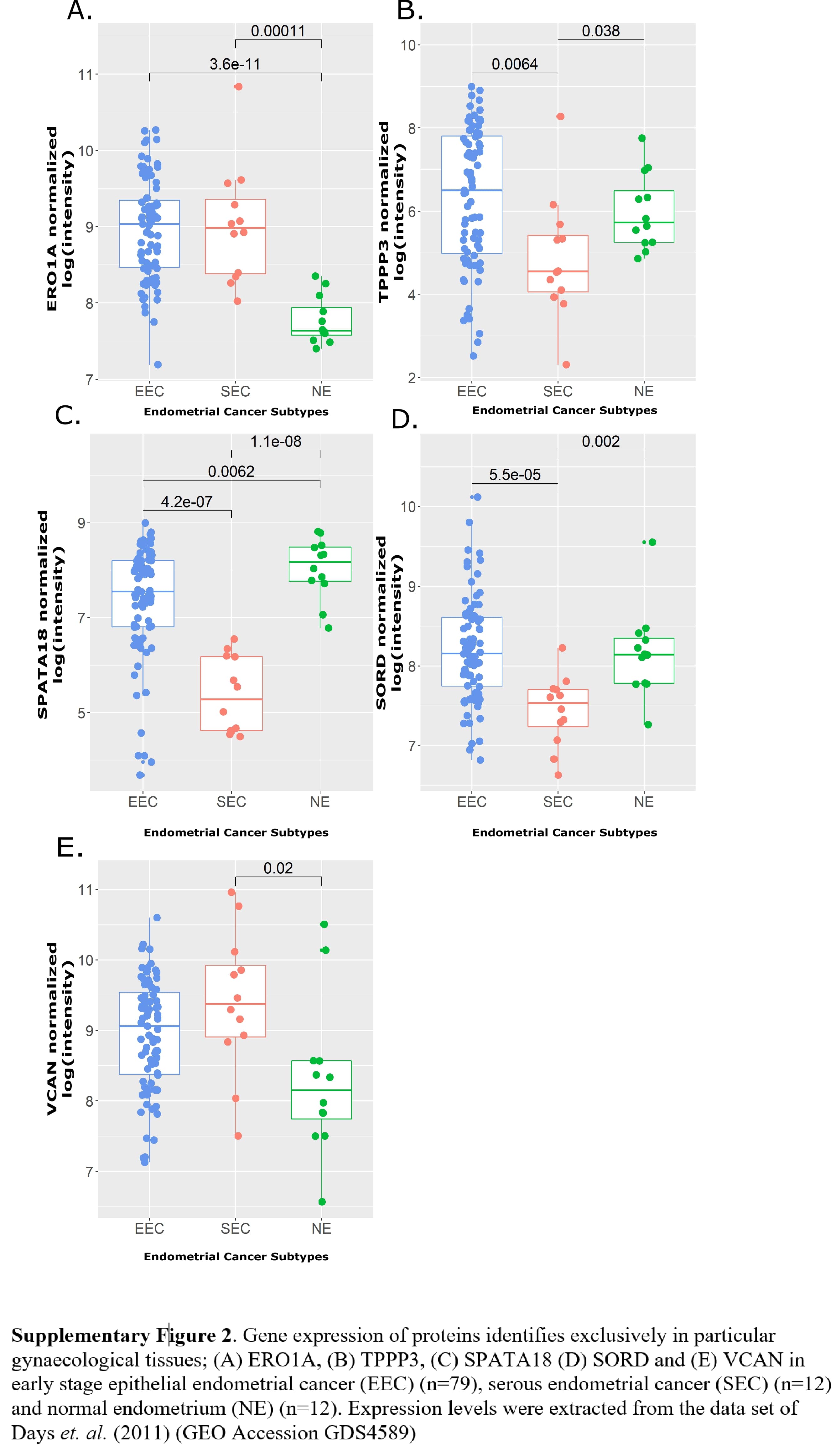

Supplement: Supplementary file 4 [file Image_2.jpeg]

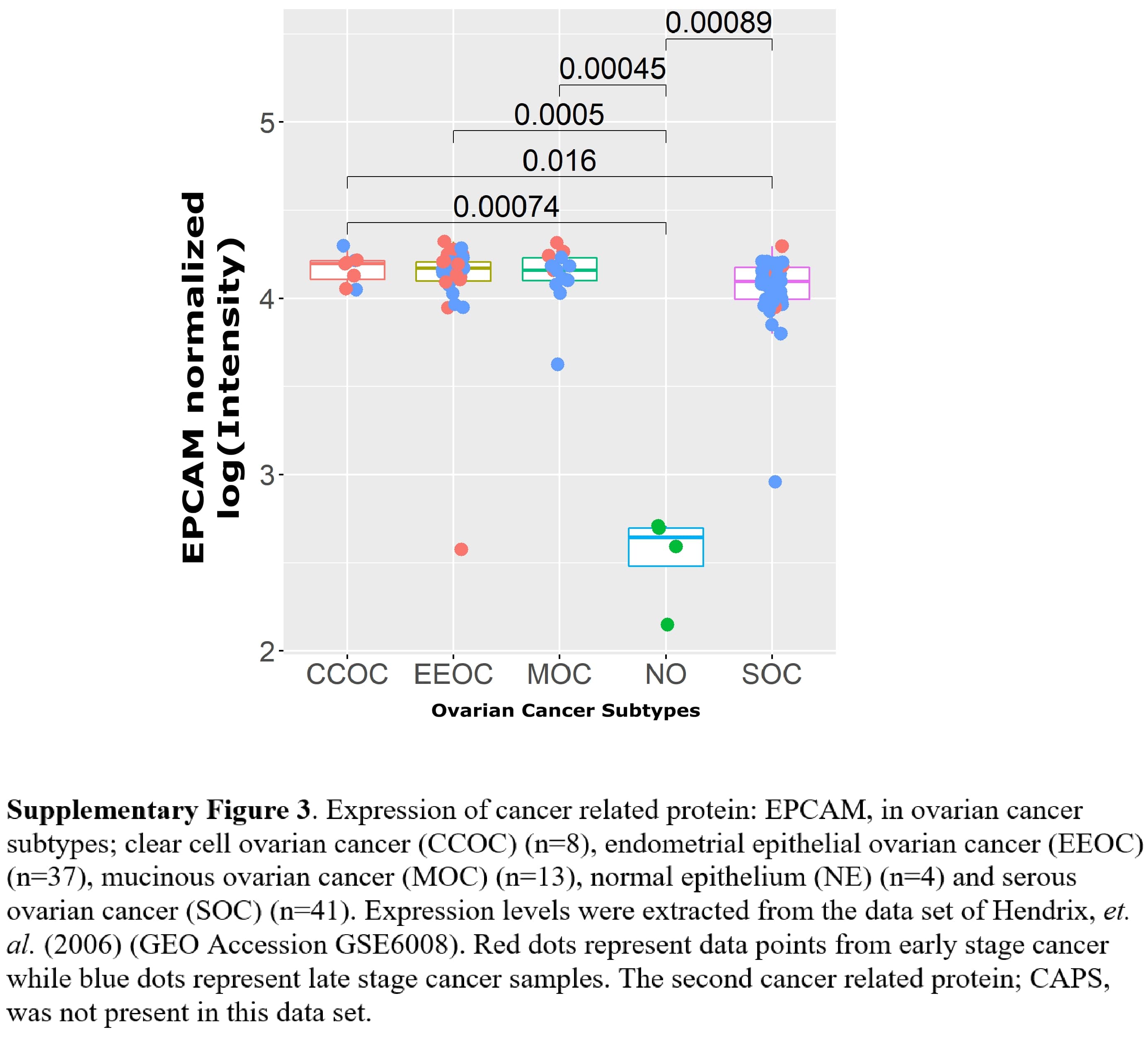

Supplement: Supplementary file 5 [file Image_3.jpeg]

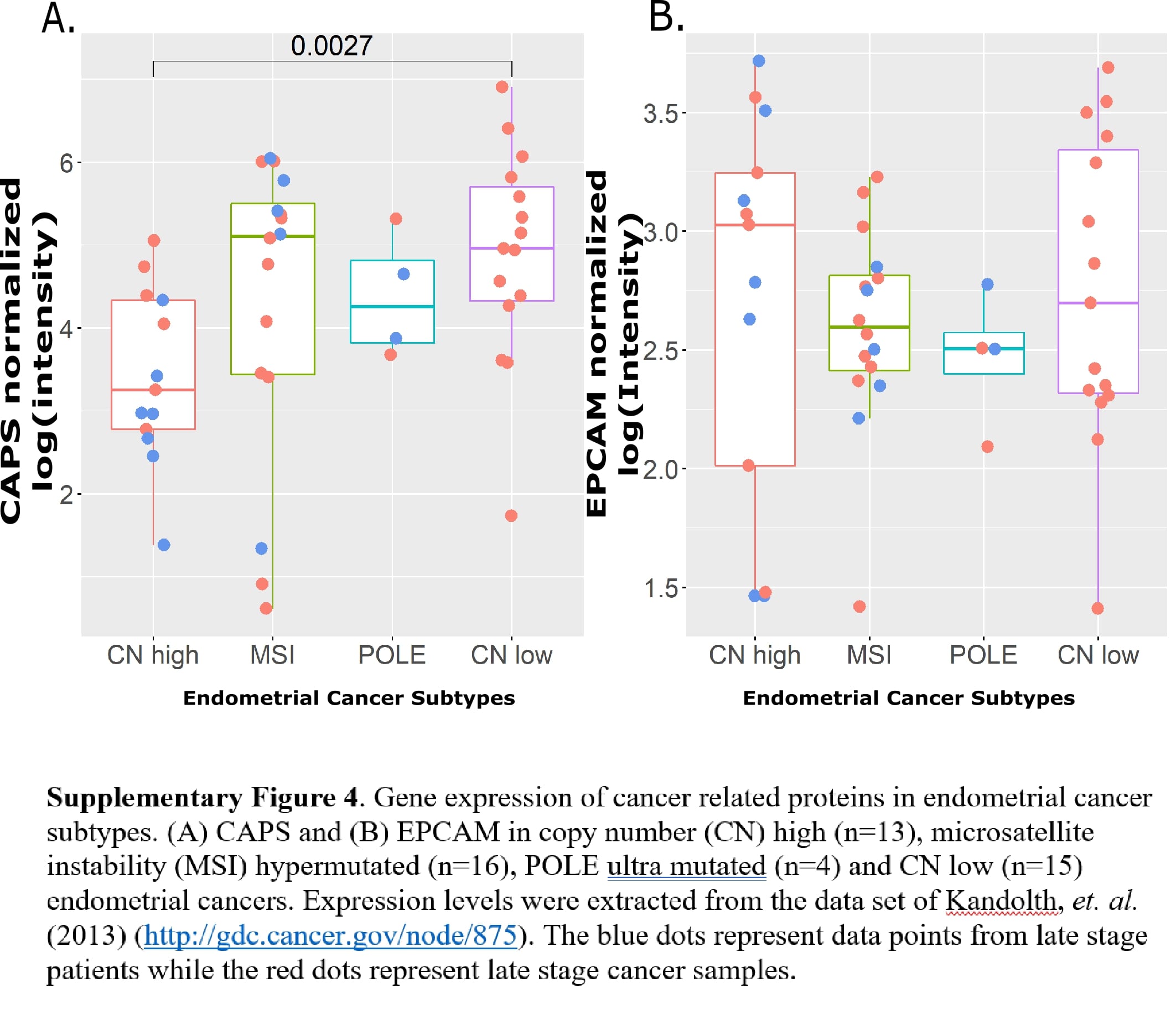

Supplement: Supplementary file 6 [file Image_4.jpeg]
